# MARCH5-dependent degradation of MCL1/NOXA complexes defines susceptibility to antimitotic drug treatment

**DOI:** 10.1038/s41418-020-0503-6

**Published:** 2020-02-03

**Authors:** Manuel D. Haschka, Gerlinde Karbon, Claudia Soratroi, Katelyn L. O’Neill, Xu Luo, Andreas Villunger

**Affiliations:** 1 Institute for Developmental Immunology, Biocenter, Medical University of Innsbruck, 6020 Innsbruck, Austria; 2 Eppley Institute for Research in Cancer and Allied Diseases, Fred and Pamela Buffet Cancer Center, University of Nebraska Medical Center, Omaha, NE 68198 USA; 3 Ludwig Boltzmann Institute for Rare and Undiagnosed Diseases, 1090 Vienna, Austria; 4 CeMM Research Center for Molecular Medicine of the Austrian Academy of Sciences, 1090 Vienna, Austria

**Keywords:** Mitochondrial proteins, Ubiquitin ligases

## Abstract

Cells experiencing delays in mitotic progression are prone to undergo apoptosis unless they can exit mitosis before proapoptotic factors reach a critical threshold. Microtubule targeting agents (MTAs) arrest cells in mitosis and induce apoptotic cell death engaging the BCL2 network. Degradation of the antiapoptotic BCL2 family member MCL-1 is considered to set the time until onset of apoptosis upon MTA treatment. MCL1 degradation involves its interaction with one of its key binding partners, the proapoptotic BH3-only protein NOXA. Here, we report that the mitochondria-associated E3-ligase MARCH5, best known for its role in mitochondrial quality control and regulation of components of the mitochondrial fission machinery, controls the levels of MCL1/NOXA protein complexes in steady state as well as during mitotic arrest. Inhibition of MARCH5 function sensitizes cancer cells to the proapoptotic effects of MTAs by the accumulation of NOXA and primes cancer cells that may undergo slippage to escape death in mitosis to cell death in the next G1 phase. We propose that inhibition of MARCH5 may be a suitable strategy to sensitize cancer cells to antimitotic drug treatment.

## Introduction

Paclitaxel (Taxol) is a well-established anticancer therapeutic used to treat ovarian, breast, and a series of other cancers since the early 1990s [1]. As a so-called microtubule targeting agent (MTA), paclitaxel interferes with microtubule dynamics and at higher concentrations leads to stabilization of microtubules [2]. Many processes like intracellular transport, cell polarization [3], and cell migration [4] rely on microtubules but interference with the mitotic spindle is thought to be crucial for the anticancer efficacy of paclitaxel. By preventing the correct function of the mitotic spindle, paclitaxel leads to chronic activation of the spindle assembly checkpoint (SAC). The SAC monitors proper chromosome attachment to the mitotic spindle to avoid premature sister chromatid segregation and as a consequence, chromosomal instability and aneuploidy. In cell culture activation of the SAC by paclitaxel arrests cells in mitosis for hours. Depending on the cell type and paclitaxel concentration used cells then either undergo apoptosis, exit mitosis without cytokinesis (also called slippage), or undergo cytokinesis with a high probability of chromosome segregation errors.

Why some cells survive mitotic arrest by slippage while other cells die within a population of seemingly identical cells remains incompletely understood but can be explained by the competing network model proposed by Gascoigne and Taylor [5]. Here, the cell cycle machinery and the apoptotic machinery are proposed to act in parallel and whichever signaling network reaches a critical threshold first defines cell fate. On the one hand, slippage is promoted through the slow degradation of Cyclin B, leading to a gradual loss of CDK1 activity which ultimately causes mitotic exit [6]. On the other hand, the rise of proapoptotic activity culminating in caspase activation determines the onset of cell death. Within the apoptotic signaling network levels of the pro-survival BCL2 family protein MCL1 assume a key role. Its degradation is regarded as a critical event, e.g., in UV-induced cell death [7], and its half-life determines the possible duration a cell can survive mitotic arrest [8]. Of note, other pro-survival proteins like BCL2, BCLX [9, 10], or BCLW [11] co-define life-span upon mitotic arrest. Remarkably, their function seems to be limited by posttranslational modifications [12] and sequestration by proapoptotic BH3-only proteins like BIM [13] rather than degradation. Regulation of MCL1 stability during mitosis is therefore an important aspect of mitotic arrest and defines the susceptibility of cancer cells to MTA treatment. Several factors have been reported to determine mitotic MCL1 stability. Phosphorylation by various kinases was suggested to target MCL1 for SCF-FBW7 [14] or APC/C [15] mediated proteasomal degradation. In addition, the proapoptotic BH3-only protein NOXA was shown to promote the mitotic degradation of MCL1 [16]. Importantly, as MCL1 needs binding to NOXA to be effectively degraded [17], hence, mitotic cell death is largely regulated by the NOXA/BIM/MCL1 axis [16, 18, 19].

The controversy still exists as to which E3-ligases control MCL1 levels in response to different types of stress including mitotic arrest [8, 20]. One E3-ligase that has been shown to regulate BCL2 family proteins is membrane associated RING finger protein 5 (MARCH5) [21, 22]. In contrast to all other E3-ligases discussed, MARCH5 is a transmembrane protein located at the outer mitochondrial membrane with a cytoplasmatic RING finger domain. Its best documented role lies in the regulation of mitochondrial fission dynamics [21, 23] but it appears to be involved in other processes like mitochondrial quality control [24], mitophagy [25], and antiviral signaling [26]. Furthermore, cells lacking MARCH5 show increased rates of cell death upon drug treatment [22, 23, 27].

Here, we addressed the role of MARCH5 in regulating MCL1 levels and show that MARCH5 deficiency increases the stability of MCL1 during early mitotic arrest. This increase in MCL1 levels is neutralized by a concomitant stabilization and accumulation of the BH3-only protein NOXA, eventually exceeding the binding capacity of endogenous MCL1. As a result, the viability of mitotically arrested cells is decreased in the absence of MARCH5. Furthermore, we show that co-depletion of NOXA, but not BIM, can restore the viability of the cells after MTA treatment. Moreover, MCL1 requires the presence of NOXA to be targeted by MARCH5 for degradation. In addition, both MCL1 and NOXA show increased rates of ubiquitination when MARCH5 is overexpressed, suggesting a direct link between MARCH5, MCL1, and NOXA. Our work identifies MARCH5 as an E3-ligase controlling sensitivity of cancer cells to MTAs by fine-tuning the turnover of MCL1/NOXA complexes. As such, inhibition of MARCH5 can be considered as a strategy to increase the efficacy of MTAs.

## Material and methods

### Cell culture

Cells were kept at 37 °C and 5% CO_2_. HeLaS3 (ATCC® CCL-2.2, gift from Erich Nigg), A549 (ATCC® CCL-185), and U2OS (ATCC® HTB-96) cells were grown in DMEM (Sigma-Aldrich, St Louis, MO, USA, D5671) supplemented with 10% FCS (Invitrogen, Waltham, MA, USA, 10270106), 1% L-Glutamine (Sigma-Aldrich, G7513), 100 U/ml Penicillin, and 100 µg/ml Streptomycin (Lonza, Basel, Switzerland, 17-602E). HCT116 cells (ATCC® CCL-247) were grown in McCoy’s 5A-Medium (Szabo Scandic, Vienna, Austria, BE12-688F) supplemented with 10% FCS, 100 U/ml Penicillin, and 100 µg/ml Streptomycin. U2OS Flag-March5 cells were a gift from Mark Wade [22]. HCT116-allBCL2KO cells were described in [28]. All cells were routinely checked for mycoplasma contamination.

### Cell synchronization and drug treatments

For analysis of cells in mitotic arrest by immunoblot, as shown in Figs. 2b, d, f, 3a–c, 4a, 5a, 6a, b, 8a, b, Supplementary Figs. 3a, 4a, b, cells were treated with 2 mM thymidine (Sigma-Aldrich, T1895) for 22 h. Cells were released by washing them twice in PBS followed by incubation in fresh medium for 9 h. 2 mM of thymidine was added for another 17 h after which the cells were washed twice with PBS and released into fresh medium with 0.5 µM paclitaxel (Sigma-Aldrich, T7191) and 10 µM Q-VD (Adooq Bioscience, Irvine, CA, USA, A14915), where applicable. Once the mitotic index reached about 30% after 11 to 12 h mitotic cells were collected by a shake-off. The remaining adherent cells were trypsinised and harvested as time point “G2”. Mitotic cells were then either harvested as time point “M”, reseeded for later mitotic time points or reseeded with addition of 20 µg/ml CHX (Sigma-Aldrich, C1988). Asynchronous cells in Fig. 8a, b were treated with 1 µg/ml doxycycline (Sigma-Aldrich, D9891) or 20 µM MG132 (Sigma-Aldrich, C2211).

### siRNA transfections

**Fig. 1:**
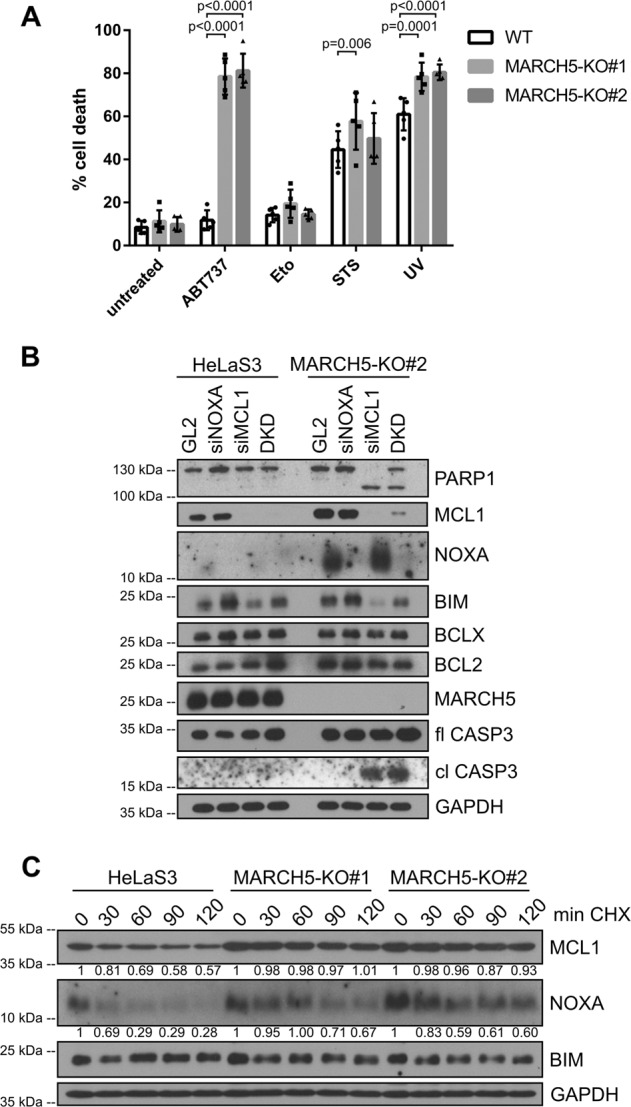
MARCH5 deficiency stabilizes both MCL1 and NOXA and increases susceptibility to specific cell death triggers. **a** Parental HeLaS3 (WT) and two clonal HeLaS3 MARCH5-KO lines created with two different small guide RNAs targeting MARCH5 (MARCH5-KO#1 and -KO#2) were treated with ABT737, etoposide (Eto), or staurosporine (STS) for 24 h. For the UV-R treatment cells were subjected to 2 mJ of UV-R and then incubated for 24 h. Cells were then harvested and propidium iodide uptake was used to measure cell death by flow cytometry. All data displayed are mean ± s.d. from five independent experiments, indicated as dots. Two-way ANOVA was used with Holm–Sidak’s multiple comparisons test to compare the WT as the control group against the two MARCH-KO lines for each treatment. All comparisons not indicated are statistically not significant (*p* > 0.05). **b** Parental HeLaS3 and HeLaS3 MARCH5-KO cells were transfected with either control siRNA targeting luciferase (GL2), NOXA and GL2 siRNA (siNOXA), MCL1 and GL2 siRNA (siMCL1) or NOXA and MCL1 siRNA (DKD) for 48 h. Cells were then harvested and prepared for immunoblot analysis. The full length (fl) and the cleaved (cl) form of caspase-3 are shown. **c** Parental HeLaS3 and two independent HeLaS3 MARCH5-KO clones were treated with Cycloheximide (CHX), harvested after the indicated time points and prepared for immunoblot analysis. Numbers below the blots show the quantification of the respective bands. Quantification was normalized to the GAPDH signal and to the untreated sample (0) of the respective genotype.

40 nM of siRNAs (final concentration) were premixed with 2 µl/ml Oligofectamine (final concentration, Invitrogen, 12252-011) in Opti-Mem (Invitrogen, 31985-054) and incubated for 20 min at room temperature. The siRNA was added to the cells 48 h before harvesting (Figs. 1b, 4b Supplementary Figs. 2b, c and 4a), 72 h before the start of live cell imaging (Figs. 5b, 5c, 8c, Supplementary Fig. 3b, c) or directly after the first washout of thymidine (Figs. 4a, 5a, 6a, 8b, Supplementary Fig. 3a). In experiments with double- or triple-knockdowns the total concentration of siRNA was 40 nM while the concentration of the single siRNAs remained constant throughout the various conditions and equal to each other. The control siRNA GL2, targeting luciferase, served as a substitute in cases with fewer targets than the maximum. The siMARCH5 #1 sequence was used where the number is not specified. All siRNAs were ordered from Microsynth, Balgach, Switzerland: GL2 (targeting luciferase) CGUACGCGGAAUACUUCGAdTdT [29], siNOXA GGUGCACGUUUCAUCAAUUdTdT [30], siMCL1 GGACUUUUAUACCUGUUAUdTdT [31], siBIM GGAGACGAGUUUAACGCUUAdTdT [32], siMARCH5 #1 GGACAGCUGUGACUUAUGGdTdT [22], siMARCH5 #2 GUAAAUUGAUGUUCAGUAGdTdT [22].

### Generation of knockout cell lines

To create MARCH5-KO in HeLa, U2OS, and A549 and BAX/BAK-KO in HeLa cells the CRISPR/Cas9 system was used. Oligonucleotides were designed with CRISPR Design (http://crispr.mit.edu, now defunct). The respective Oligonucleotides were cloned into the lentiCRISPR version 2 backbone (a gift from Feng Zhang; Addgene plasmid number 52961) according to the Feng Zhang protocol. To create BAX/BAK double deficient HeLa cells the BAX oligonucleotide was cloned into a modified version of the lentiCRISPR version 2 where the puromycin resistance had been exchanged for a blasticidin resistance (a gift from Sebastian Herzog). All plasmids were verified by sequencing. Cells were transduced with lentiviral supernatants and selected for genomic integration with puromycin or blasticidin. HeLa single cell clones were isolated by seeding the cells into a 96 well plate with a density of 0.1 cells per well. Knockout clones were confirmed using immunoblot. The guide sequences were as follows: BAX: CGAGTGTCTCAAGCGCATCG, BAK: GCCATGCTGGTAGACGTGT, MARCH5-KO#1: TAATGGTCGGCTCTATCTAT, and MARCH5-KO#2: AGGCAAGATGATTCGCTGGG.

### Retroviral transduction of cell lines

For the reconstitution of the HCT116-allBCL2KO cell line with NOXA and/or MCL1 expression human NOXA or NOXA-L29E cDNA was cloned into a pMIG (MSCV IRES eGFP) vector. Human MCL1 cDNA was cloned into a modified version of the pMIG vector where the eGFP was exchanged for a dsRed-Express2 (a gift from Sebastian Herzog). Retroviral supernatant was used to transduce HCT116-allBCL2KO cells. Transduced cells were identified by their fluorescence and sorted with a FACS Aria III (BD Biosciences, Franklin Lakes, NJ, USA) to obtain a uniform population.

### Cell lysis and immunoblot

Cells were trypsinised, washed in PBS and incubated with lysis buffer (50 mM Tris pH 8.0, 150 mM NaCl, 0.5% NP-40, 50 mM NaF, 1 mM Na_3_VO_4_, 1 mM PMSF, one tablet protease inhibitors (EDTA free, Roche, Basel, Switzerland, 11873580001) per 10 ml and 30 mg/ml DNaseI (Sigma-Aldrich, DN-25)) for 30 min on ice. After clearing of insoluble debris through 15 min centrifugation at 21,000 × *g* and 4 °C protein concentration was measured by Bradford analysis (Bio-Rad, Hercules, CA, USA 500-0006). After addition of SDS loading buffer (final concentration 50 mM Tris, 2% SDS, 0.1% bromphenol blue, 10% glycerol, 86 mM ß-mercaptoethanol) samples were boiled at 95 °C for 5 min. 40–60 µg of total protein were run on a SDS-PAGE and blotted on a nitrocellulose membrane (GE Healthcare Life Science, 10600004). For denatured immunoprecipitation and TUBE experiments the membrane was autoclaved after transfer for 20 min. Membranes were blocked in 5% milk (Sigma-Aldrich, 70166) in PBS-T for 1 h and incubated with the primary antibodies diluted in 5% BSA (Sigma-Aldrich, 12659-M) in PBS-T overnight at 4 °C. Membranes were washed five times in PBS-T for 5 min. After secondary antibody incubation in 5% milk in PBS-T for 1 h at room temperature membranes were washed again five times in PBS-T for 5 min. Signal detection was achieved by incubation with ECL (Biozym, 541006) and chemiluminescence films (GE Healthcare Life Science, Chicago, IL, USA, 28906837 or Agfa, Mortsel, Belgium, 34YAX). Antibodies used were: rabbit anti MARCH5 (Millipore, Burlington, MA, USA, 06–1036, 1:500), mouse anti NOXA (clone 114C307, Rockland Immunochemicals, Limerick, PA, USA, 200-301-H98, 1:500), rabbit anti MCL1 (Santa Cruz Biotechnology, Dallas, TX, USA, sc-819, 1:1000, discontinued), rabbit anti PARP1 (Cell Signaling, Danvers, MA, USA, #9542, 1:1000), rabbit anti CASP3 (Cell Signaling #9662, 1:1000), rabbit anti BIM (Enzo Life Sciences, Farmingdale, NY, USA, ADI-AAP-330-E, 1:500), mouse anti Ubiquitin (clone P4D1, Cell Signaling #3936, 1:1000), rabbit anti GAPDH (clone 14C10, Cell Signaling #2118, 1:5000), mouse anti HSP 90 (clone F8, Santa Cruz Biotechnology, sc-13119, 1:1000), rabbit anti BCLX (clone 54H6, Cell Signaling #2764, 1:1000), mouse anti BCL2 (clone S100, gift from Andreas Strasser, 1 µg/ml), goat anti rabbit IgG-HRP (Dako, Glostrup, Denmark, P0448, 1:5000), and rabbit anti mouse-IgG-HRP (Dako P0161, 1:5000). Western blot quantification was done by densitometric analysis using the gel analysis function of Fiji.

### Immunoprecipitation

The denaturing immunoprecipitation (Fig. 7a) was adapted from [21]: Harvested cells were resuspended in denaturing buffer (1% SDS, 5 mM EDTA, 10 mM β-mercaptoethanol) and incubated at 100 °C for 10 min. After centrifugation at 21,000 × *g* for 5 min to clear the lysate of insoluble debris the supernatant was diluted by addition of ten times its volume of immunoprecipitation buffer (20 mM Tris-HCl, pH 7.5, 150 mM NaCl, 1 mM EDTA, 0.5% NP-40, 5 mM N-ethylmaleimide, and protease inhibitors) and protein concentration was measured by Bradford analysis. For non-denaturing immunoprecipitation (Supplementary Fig. 4a, b) cells were lysed as described in “Cell lysis and immunoblot” until protein concentration was measured. 60 µg of protein was taken as the “input” sample.

Protein A beads (Bio-Rad, 156-0006) were crosslinked with MCL1 (Santa Cruz Biotechnology sc-819, discontinued), NOXA (Cell Signaling #14766), or MARCH5 (Millipore 06–1036) antibody by rotation of 1 µg (1 µl for NOXA) of antibody with 20 µl of slurry beads per condition at 4 °C for 2 h followed by a 30 min incubation in coupling buffer (200 mM Na_2_B_4_O_7_ pH 9.0, 20 mM Dimethyl Pimelimidate) at room temperature. The coupling reaction was quenched by incubation of the beads with 200 mM Ethanolamine, pH 8.0 for 2 h at room temperature.

After equilibration of the beads in immunoprecipitation buffer for denatured lysates or immunoblot lysis buffer for non-denatured lysates the coupled beads were added to 600–1000 µg of lysates and incubated on a rotating wheel at 4 °C overnight. After centrifugation at 100 × *g* for 1 min to sediment the beads a volume corresponding to 60 µg of protein was taken as the “unbound” sample. After two washes with immunoprecipitation or immunoblot buffer and three washes with PBS beads were resuspended in SDS loading buffer and boiled at 95 °C for 5 min.

### TUBE assay

To isolate ubiquitinated proteins TUBE 2 agarose (Tebu-bio, Le Perray en Yvelines, France, UM402-1M) with an adapted version of the manufacturer’s instructions was used. Briefly, cells were harvested by trypsinisation and lysed in TUBE lysis buffer (50 mM Tris-HCl, pH 7.5, 0.15 M NaCl, 1 mM EDTA, 1% NP-40, 10% glycerol) for 30 min on ice. After clearing of insoluble debris by centrifugation at 21,000 × *g* for 15 min the protein concentration was measured by the Bradford assay. 60 µg of protein was taken as the “input” sample while 750 µg of protein per condition was incubated with 20 µl of TUBE 2 agarose on a rotating platform at 4 °C overnight. After centrifugation at 3000 × *g* for 5 min to sediment the beads a volume corresponding to 60 µg of protein was taken as the “unbound” sample. The beads were then washed with TBS-T (20 mM Tris-HCl, pH 8.0, 0.15 M NaCl, 0.1% Tween-20) three times. After resuspension in SDS loading buffer the beads were boiled at 95 °C for 5 min.

### Live cell imaging

**Fig. 2:**
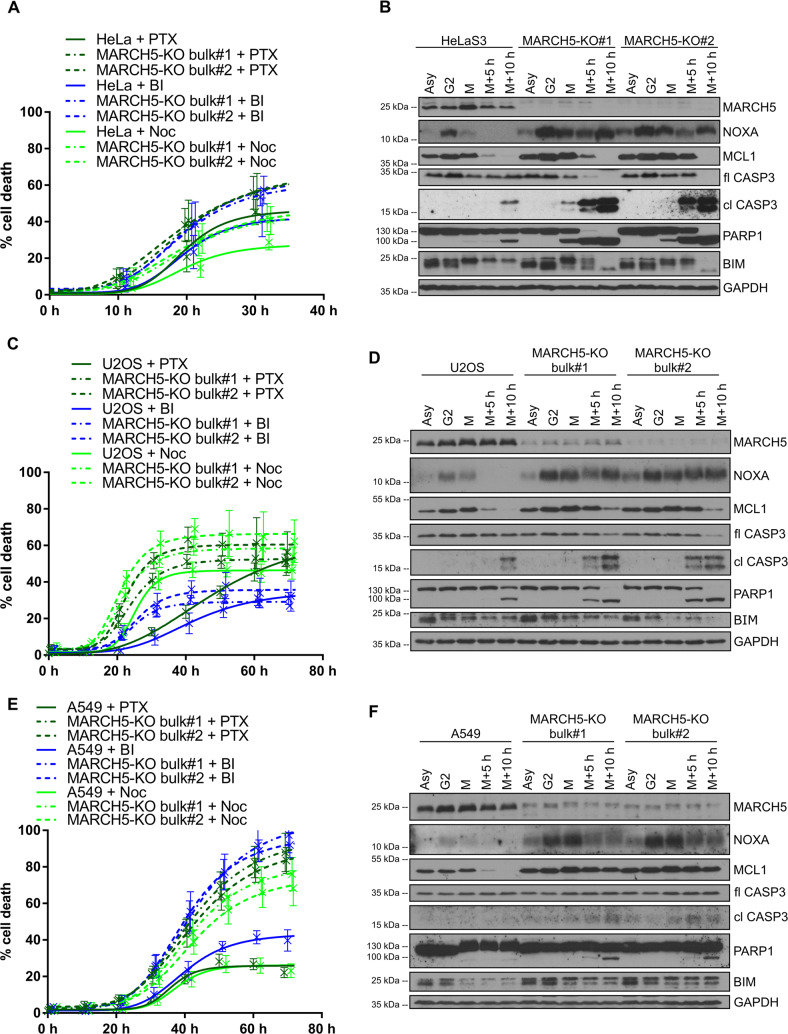
Lack of MARCH5 sensitizes to cell death during mitotic arrest where MCL1 and NOXA are stabilized. **a** Parental HeLaS3 and two HeLaS3 MARCH5-KO bulks generated with two different small guide RNAs were treated with paclitaxel (PTX), nocodazole (Noc), or BI2536 (BI) immediately followed by live cell imaging. Addition of propidium iodide to the medium allowed the automated assessment of dead cells using the IncuCyte software. A nonlinear regression curve of the percentage of dead cells in relation to the total number of cells present at the beginning of the imaging of four independent experiments is shown. The mean and s.d. of exemplary data points are also shown. **b** Parental HeLaS3 and two independent HeLaS3 MARCH5-KO clones were either left asynchronous (Asy) or synchronized by double thymidine block, released into paclitaxel and harvested at the respective time points. Samples were then prepared for immunoblot analysis. For caspase-3 the full length (fl) and cleaved (cl) form is shown. **c** Same as in a). Three independent experiments with U2OS cells and two U2OS MARCH5-KO bulks generated with two different small guide RNAs. **d** Same as in b) with U2OS cells and two U2OS MARCH5-KO bulks generated with two different small guide RNAs. **e** Same as in a). Three independent experiments with A549 cells and two A549 MARCH5-KO bulks generated with two different small guide RNAs. **f** Same as in b) with A549 cells and two A549 MARCH5-KO bulks generated with two different small guide RNAs.

For experiments shown in Fig. 2a, c, e cells were treated with 0.5 µM paclitaxel (Sigma-Aldrich, T7191), 1 µM nocodazole (Sigma-Aldrich, M1404), or 0.1 µM BI2536 (Selleck Chemicals, Houston, TX, USA, S1109) together with 1 µg/ml propidium iodide (Sigma-Aldrich, 81845) and imaged every 20 min in an IncuCyte S3 microscope (Sartorius, Göttingen, Germany) using the ×10 objective. Four positions per condition were imaged. The analysis function of the IncuCyte software was used to count propidium iodide positive cells. To calculate the ratio of cell death the total amount of cells present at the beginning of the experiment was counted manually using Fiji.

For experiments shown in Figs. 5b, 8C and Supplementary Fig. 3b cells were first synchronized with 2 mM thymidine for 24 h. After washing the cells twice with PBS they were released into L15K medium together with 0.5 µM paclitaxel (Sigma-Aldrich, T7191). Image acquisition without binning was done every 5 min with a DMi8 (Leica Microsystems, Wetzlar, Germany) with a ×10 objective, a OrcaFlash 4 camera (Hamamatsu Photonics, Hamamatsu, Japan) and an environmental chamber set to 37 °C. Two positions per condition were imaged. For analysis a total of 100 cells per treatment (50 cells per position) entering mitosis were randomly selected and mitotic duration, mitotic cell fate and duration of the following interphase were assessed manually according to cell morphology using Fiji.

For experiments shown in Supplementary Fig. 1a, b, c cells were treated with 10 µM S63845 (Selleck Chemicals, S8383), 1 µM ABT737 (Selleck Chemicals, S1002), or 1 µM ABT737 plus 1 µM S63845. All cells were also treated with 1 µg/ml propidium iodide (Sigma-Aldrich, 81845). Cells were imaged every hour in an IncuCyte S3 microscope (Sartorius) using the ×10 objective. Four positions per condition were imaged. The analysis function of the IncuCyte software was used to count propidium iodide positive cells which were normalized to the area covered by the cells at the start of imaging. The covered area was also determined by the analysis function of the IncuCyte.

### Flow cytometry

For Fig. 1a cells were treated with one exposure to 2 mJ UV radiation, 100 nM staurosporine (Sigma-Aldrich, S6942), 10 µM ABT737 (Selleck Chemicals, S1002), or 20 µM etoposide (Sigma-Aldrich E1383) for 24 h. Cells were harvested, washed with PBS and resuspended in PBS with 1 µg/ml propidium iodide (Sigma-Aldrich, 81845). The samples were measured with an Attune NxT flow cytometer (Thermo Fisher Scientific, Waltham, MA, USA). The acquired data was analyzed with FlowJo software (version X, FlowJo LLC, Ashland, OR, USA).

### Statistical analysis

For statistical analysis two-way (Fig. 1a) or one-way (Figs. 5c, 8c, Supplementary Fig. 3c) ANOVA with the Holm–Sidak’s multiple comparisons test was calculated in Prism 7 (GraphPad Software, San Diego, CA, USA). The nonlinear regression shown in Fig. 2a, c, e, Supplementary Fig. 1a–c was calculated in Prism 7 as a four-parameter dose-response curve (variable slope). The number of biological replicates was pre-determined and is given in the legends of the individual experiments.

## Results

### MARCH5 controls cell survival and turnover of NOXA/MCL1 complexes

Knockdown of the E3-ligase MARCH5 has been described to sensitize U2OS osteosarcoma as well as HCT116 colorectal cancer cells to the effect of BCL2 inhibition [22]. Indeed, when HeLa MARCH5-KO cells were treated with the BH3-mimetic ABT737 or UV-radiation (UV-R), they were more susceptible to cell death than parental cells (Fig. 1a, Supplementary Fig. 1a). In contrast, this was not observed with the DNA damaging agent etoposide or the pan-kinase inhibitor staurosporine (STS). Notably, in both conditions, ABT737 or UV-R, degradation of the pro-survival protein MCL1 has been described to be of particular importance for induction or enhancement of cell death [7, 33]. Of note, knockout of MARCH5 also sensitized A549 lung adenocarcinoma and U2OS osteosarcoma cells to ABT737 treatment, indicating that the observed sensitization was not limited to HeLa cells (Supplementary Fig. 1b, c). Intriguingly, we also noted that levels of MCL1, but not BCL2 or BCLX, were increased in the absence of MARCH5 (Fig. 1b, Supplementary Fig. 2a, b).

To understand if the increase in MCL1 may be protective for cells lacking MARCH5, we conducted siRNA-mediated knockdown experiments in HeLa cells. We noted that PARP1 cleavage, a marker for caspase activation, occurred spontaneously after knockdown of MCL1 in HeLa MARCH5-KO cells but not in parental cells (Fig. 1b). As NOXA levels are drastically increased when MARCH5 is depleted, we were wondering if NOXA is responsible for the cell death caused by MCL1 knockdown. To test this we depleted NOXA together with MCL1 in HeLa MARCH5-KO cells and could indeed observe a reduction in PARP1 cleavage (Fig. 1b). However, when conducting similar experiments in A549, as well as U2OS cells (Supplementary Fig. 2a, b), we failed to see induction of cell death by knockdown of MCL1 alone in these cells lacking MARCH5. To exclude a lack of effect by insufficient knockdown efficiency we switched to chemical inhibition of MCL1 using the BH3-mimetic S63845. S63845 treatment that was most potent in inducing cell death in HeLa and less potent in U2OS cells failed to kill A549 cells (Supplementary Fig. 1a–c). Importantly, HeLa and U2OS were sensitized to MCL1 inhibition by MARCH5 depletion (Supplementary Fig. 1a, b). A549 cells, where NOXA levels were hardly detectable in unsynchronized cells even after MARCH5-KO (Supplementary Fig. 2b), however, were not sensitized to S63845 when MARCH5 was depleted. This suggests that these cells do not depend on MCL1 for survival, as NOXA levels are extremely low. Consistent with this idea, A549 cells were only killed by a combination of ABT737 and S63845, the combination of which was most effective again in HeLa over U2OS cells (Supplementary Fig. 2a–c).

We wondered if the increase in MCL1 and NOXA levels seen in all MARCH5-KO cell lines tested was due to increased protein stability. Thus, we blocked protein translation using cycloheximide (CHX). Indeed, degradation of both proteins was reduced in MARCH5-KO cells (Fig. 1c). Together, this suggests a role for MARCH5 as an E3-ligase for MCL1 and NOXA and confirms that the loss of MARCH5 sensitizes cells to defined cell death triggers that involve the NOXA/MCL1 signaling axis.

### MARCH5 contributes to MCL1/NOXA co-degradation during mitotic arrest

Next, we wondered if depletion of MARCH5 could sensitize cells to MTA treatment that depends on the NOXA/MCL1 signaling axis [16]. Hence, we treated HeLa, U2OS and A549 WT, and MARCH5-KO cells with paclitaxel, the microtubule destabilizer nocodazole or the PLK1 inhibitor BI2536. PLK1 inhibition does not interfere with microtubule dynamics but leads to the formation of monopolar mitotic spindles and therefore activates the SAC [34]. MARCH5-KO cells died faster to all three mitotic inhibitors when compared with WT cells (Fig. 2a, c, e). To address the role of MARCH5 in mitotic cell death further, we synchronized HeLa, U2OS and A549 WT, and MARCH5-KO cells and treated them with paclitaxel. Indeed, as judged by the appearance of cleaved caspase-3 as well as PARP1 all MARCH5-KO cell lines were clearly more sensitive to mitotic cell death than WT cells, including the rather resistant A549 cells (Fig. 2b, d, f).

We and others have shown that cell death caused by paclitaxel depends on MCL1 which itself is antagonized by NOXA [16, 19]. Of note, MARCH5 depletion led to the accumulation of MCL1 and NOXA in asynchronous as well as G2 cells, suggesting that MARCH5 controls NOXA/MCL1 levels throughout the cell cycle. During extended mitotic arrest in HeLa cells, however, MCL1 was still degraded in the absence of MARCH5 with kinetics similar to WT cells. Strikingly, in the rather cell death resistant U2OS and A549 cells MCL1 levels were clearly more stable in MARCH5-KO cells than WT. In contrast, NOXA levels remained nearly stable throughout the duration of mitotic arrest in MARCH5-KO HeLa, U2OS and A549 cells.

**Fig. 3:**
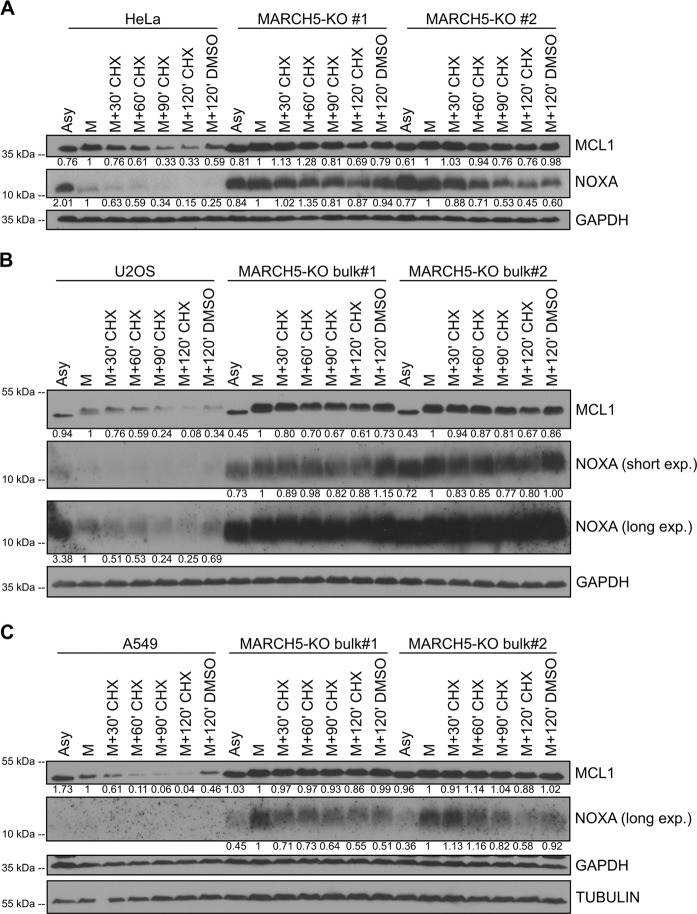
Lack of MARCH5 stabilizes MCL1 and NOXA early during mitotic arrest. **a** Parental HeLaS3 and two independent HeLaS3 MARCH5-KO clones were either left asynchronous (Asy) or synchronized by double thymidine block and released into paclitaxel. Once cells were mitotic (M), they were treated with Cycloheximide (CHX) or solvent control (DMSO) for the indicated times in minutes. Numbers below the blots show the quantification of the respective bands. Quantification was normalized to the GAPDH signal and to the early mitotic arrest sample without CHX (M) of the respective genotype. **b** Same as in a) with U2OS cells and two U2OS MARCH5-KO bulks generated with two different guide RNAs. For NOXA a short and a long exposure are shown. **c** Same as in a) with A549 cells and two A549 MARCH5-KO bulks generaed with two different guide RNAs.

**Fig. 4:**
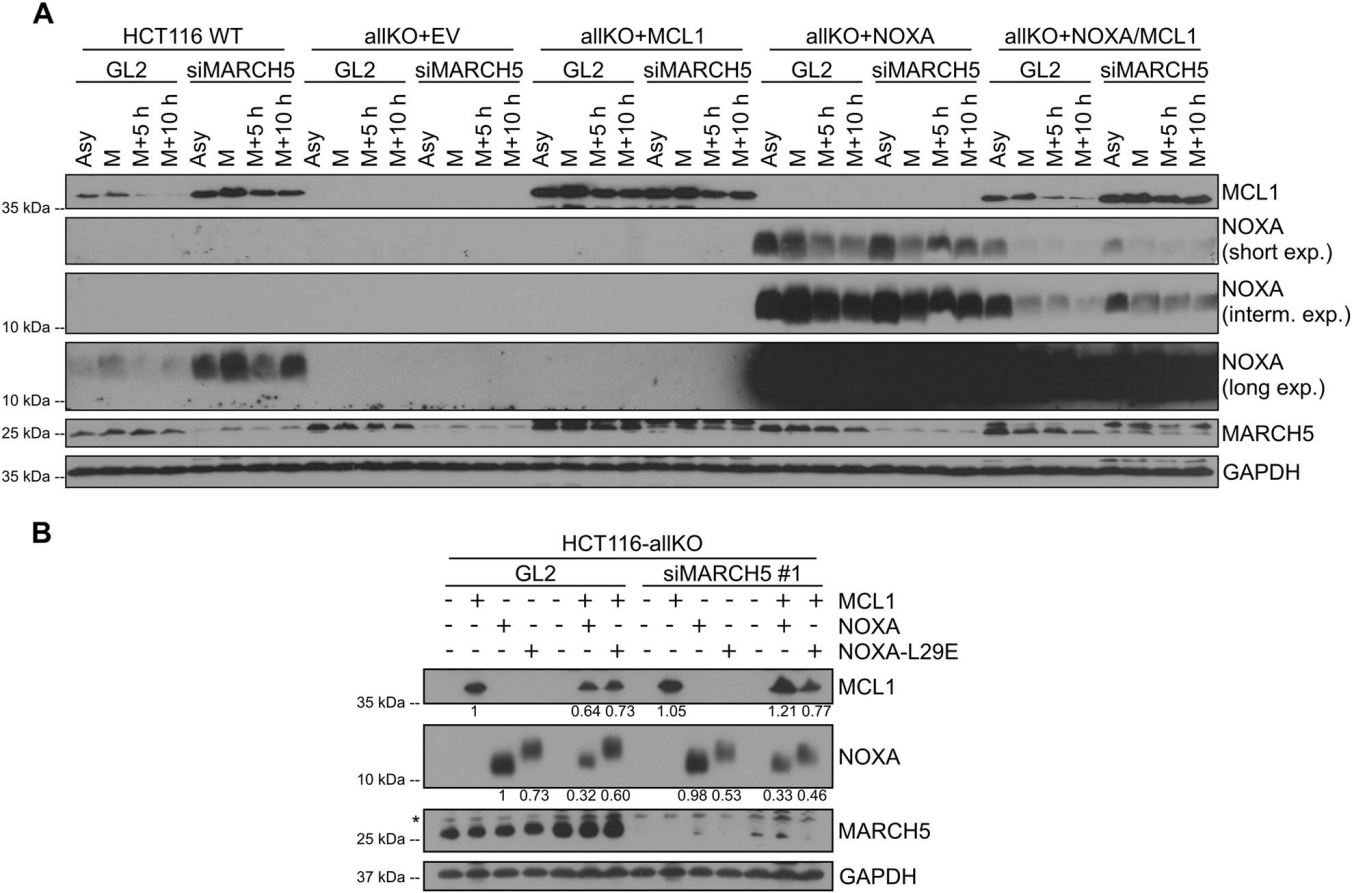
MCL1 is only stabilized in the absence of MARCH5 if NOXA is present and able to bind MCL1. **a** Parental HCT116 (WT) and HCT116-allBCL2KO (allKO) cells transduced with empty vector controls (EV), NOXA, MCL1 or both NOXA and MCL1 were transfected with the indicated siRNAs. The cells were left asynchronous (Asy) or synchronized by double thymidine block, released into paclitaxel and harvested at the indicated time points once cells entered mitotic arrest (M). For NOXA a short, an intermediate and a long exposure are shown. **b** HCT116-allBCL2KO (HCT116-allKO) cells transduced with expression vectors carrying MCL1, NOXA, NOXA-L29E or no cDNA were transfected with the indicated siRNAs. After 48 h cells were prepared for immunoblot analysis. Numbers below the blots show the quantification of the respective bands. Quantification was normalized to the GAPDH signal and to the single-transduced MCL1 or NOXA sample transfected with the control siRNA (GL2). The asteriks indicates unspecific bands in the MARCH5 blot.

As multiple E3-ligases have been implicated to degrade MCL1 in mitosis we next investigated the effect of MARCH5 on the stability of NOXA and MCL1 during mitotic arrest. Hence, we again used CHX to block protein synthesis. Both MCL1 and NOXA were more stable in the absence of MARCH5 in HeLa and U2OS during mitotic arrest (Fig. 3a, b). Notably, in A549 we could only demonstrate the stabilization of MCL1 since we were unable to detect NOXA in sufficient quality to be able to compare it to the MARCH5-KO cells. Since we have previously shown that the stability of MCL1 during mitotic arrest is dependent on NOXA, we wondered if there is a co-dependence for protein turnover by MARCH5. To test this we used HCT116 cells devoid of all BCL2 family members [28] (HCT116-allBCL2KO). We reconstituted these cells with exogenous expression of NOXA, MCL1 or both. Next, we depleted MARCH5 with siRNA and compared the levels of NOXA and MCL1 during mitotic arrest against those found in control transfected cells and parental HCT116 cells. In HCT116 WT cells, reduction of MARCH5 led to the expected increase in MCL1 and NOXA levels in asynchronous as well as paclitaxel treated cells (Fig. 4a). However, in both the single MCL1 and the single NOXA reconstituted cell line, levels of neither MCL1 nor NOXA changed dramatically after MARCH5 depletion when compared with the control transfection. Critically, when both proteins were reconstituted together, MCL1 levels increased after MARCH5 depletion similar to what was observed in WT cells (Fig. 4a). Exogenous NOXA levels, however, clearly exceeded those of the endogenous protein and remained mostly unaffected by the knockdown of MARCH5. Interestingly, when the NOXA-L29E mutant carrying a point-mutation preventing binding to MCL1 [35] was co-expressed with MCL1 no increase in MCL1 levels in MARCH5 depleted cells was observed (Fig. 4b). Together, this suggests that degradation of MCL1 by MARCH5 depends on binding to NOXA, but degradation of NOXA may be promoted by additional E3-ligases if MCL1 levels become limited, or can simply no longer be degraded.

### NOXA sensitizes to mitotic cell death in the absence of MARCH5

**Fig. 5:**
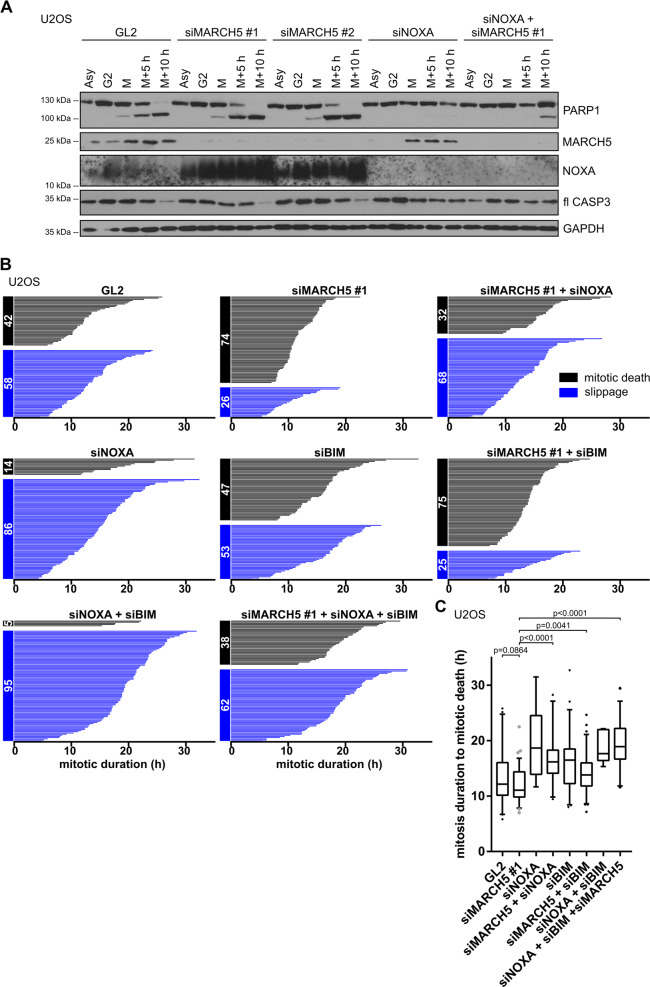
The increase in cell death caused by depletion of MARCH5 is NOXA dependent. **a** Immunoblots of U2OS cells transfected with either a control siRNA targeting luciferase (GL2), GL2 and MARCH5 siRNA (siMARCH5 #1), GL2 and a different MARCH5 siRNA (siMARCH5 #2), GL2 and NOXA siRNA (siNOXA) or NOXA and MARCH5 #1 siRNA. Cells were left asynchronous or synchronized by double thymidine block, released into paclitaxel and harvested at the indicated time points for immunoblot analysis. The full length (fl) form of caspase-3 is shown. **b** Cell fate profiles of U2OS cells arrested in mitosis. Each horizontal bar indicates the duration of mitotic arrest of a single cell. Numbers to the left of the fate profiles indicated how many cells died or underwent slippage at the end of mitotic arrest. U2OS cells were transfected with the indicated siRNAs, synchronized by a single thymidine block followed by release into paclitaxel and the start of live cell imaging. **c** Box blots with 5–95% whiskers of the mitotic duration of U2OS cells that died during mitotic arrest (black bars in fate profiles) shown in b). One-way ANOVA was used with Holm–Sidak’s multiple comparisons test to compare the mitotic durations of the siMARCH5 sample against all other knockdowns. The *p*-values for selected populations are shown with *p* < 0.05 considered statistically significant.

Next, we investigated if the increase in cell death during mitotic arrest is dependent on NOXA. We therefore depleted NOXA, MARCH5 or both with siRNAs in HeLa or U2OS cells and treated them with paclitaxel. Knockdown of MARCH5 increased caspase activity as judged by increased PARP1 cleavage in both cell lines (Fig. 5a, Supplementary Fig. 3a). NOXA-KD, on the other hand completely abolished PARP1 cleavage and prevented its occurrence almost completely when MARCH5 was co-depleted in U2OS cells (Fig. 5a). In HeLa cells the same trends were observed (Supplementary Fig. 3a).

In this type of bulk analysis cells could have died during mitotic arrest or after cells underwent slippage. We thus analyzed mitotic duration, mitotic death, and slippage using live cell imaging of U2OS cells, as these cells are more slippage-prone [36]. We found that in paclitaxel treated cells MARCH5-KD increases the number of cells dying during mitotic arrest (Fig. 5b). This increase in mitotic cell death could be prevented by co-depletion of NOXA. Notably, NOXA knockdown alone conferred a prominent increase of cells that exit mitotic arrest via slippage, consistent with the competing network hypothesis [5]. We repeated the same experiment using HeLa cells. However, as these cells die preferentially in mitosis after paclitaxel treatment NOXA knockdown did not affect the proportion of cells dying but instead led cells to survive longer until they succumbed to mitotic death (Supplementary Fig. 3b, c). Of note, MARCH5 knockdown shortened the duration of mitotic arrest and abrogated the residual slippage seen in HeLa cells completely. Combining both NOXA and MARCH5 siRNAs the duration of mitotic arrest prior to cell death was again comparable to the one of the control siRNA.

Since we and others previously noted additive effects of NOXA with BIM in mitotic cell death [16, 18] we investigated if BIM depletion has a similar effect on MARCH5 dependent sensitization to mitotic cell death of U2OS cells. Of note, BIM protein levels were not increased by MARCH5 depletion during steady state or mitotic arrest (Fig. 2b, d). Accordingly, BIM knockdown could not increase the rate of cells surviving MARCH5 co-depletion (Fig. 5b). Consistent with this, BIM/NOXA/MARCH5 triple-knockdown failed to increase resistance to mitotic death compared with NOXA/MARCH5 double-depletion. Nevertheless, BIM-depletion did cause an extension of mitotic duration in those cells that ultimately died during mitotic arrest (Fig. 5c).

In HeLa cells similar observations in regards to the role of BIM were made. BIM knockdown alone or in conjunction with NOXA extended the mitotic duration prior to cell death (Supplementary Fig. 3c), corroborating our previous findings [16]. However, BIM was unable to provide statistically significant protection against depletion of MARCH5. Similarly, BIM/NOXA/MARCH5 triple-knockdown could not confer better cell death protection than NOXA/MARCH5 double-knockdown. Taken together this suggests that the sensitization to mitotic cell death caused by MARCH5 deficiency is linked to increased NOXA levels and is not affected by BIM.

### Caspase activation limits NOXA degradation but enhances MCL1 degradation

**Fig. 6:**
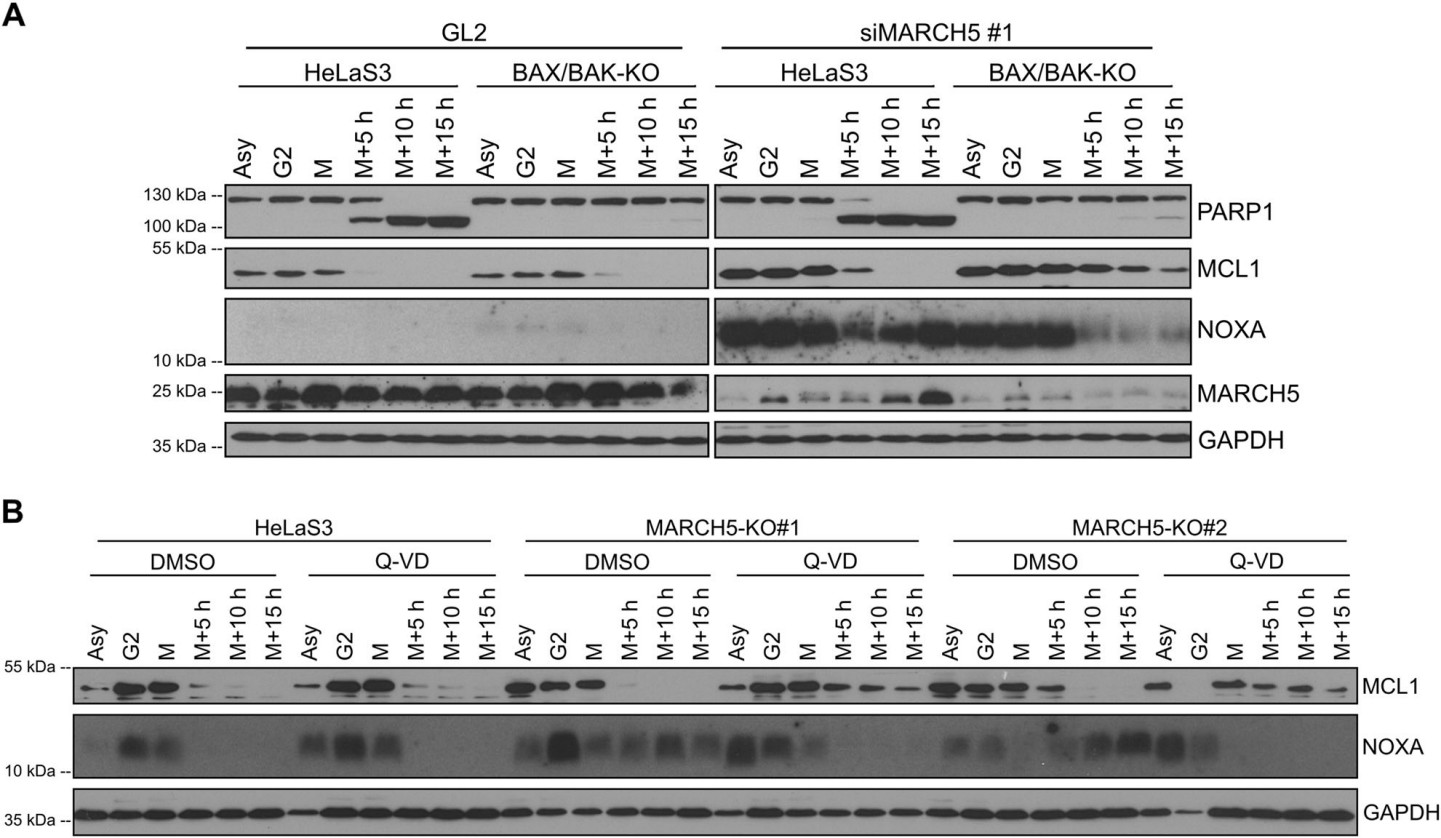
Cell death inhibition in cells lacking MARCH5 increases MCL1 but decreases NOXA levels. **a** Parental HeLaS3 and HeLa BAX/BAK knockout cells were transfected with control siRNA (GL2) or MARCH5 siRNA. Cells were left asynchronous (Asy) or synchronized by a double thymidine block, released into paclitaxel and harvested at the indicated times. Samples were then prepared for immunoblot analysis. **b** Parental HeLaS3 cells and two independent MARCH5-KO clones were left asynchronous (Asy) or synchronized by double thymidine block, released into paclitaxel together with DMSO or Q-VD and harvested at the indicated time points.

Since MARCH5 deficiency increases cell death rates during mitotic arrest we aimed to separate effects due to loss of MARCH5 from effects occurring secondary to apoptosis initiation. We therefore analyzed HeLa BAX/BAK double-knockout cells, depleted MARCH5 using siRNAs and compared NOXA and MCL1 levels during mitotic arrest to those seen in parental HeLa. Surprisingly, neither the complete degradation of MCL1 nor stabilization of NOXA was observed in the later stages of mitotic arrest in BAX/BAK double-knockout cells when MARCH5 was depleted (Fig. 6a), suggesting that caspase-activation impacts NOXA/MCL1 co-degradation. To confirm this hypothesis, we used the pan-caspase inhibitor Q-VD as another measure to block apoptosis in HeLa cells lacking MARCH5. Consistently, cells lacking MARCH5 treated with Q-VD showed an increased stability of MCL1 and a more effective degradation of NOXA during mitotic arrest when compared with DMSO treated cells (Fig. 6b). Together this suggests that in MARCH5 depleted cells caspase activation affects MCL1 turnover, possibly by inactivating its translation or by direct cleavage, while NOXA only accumulates as soon as MCL1 is lost.

### MARCH5 promotes ubiquitination of MCL1 and NOXA

**Fig. 7:**
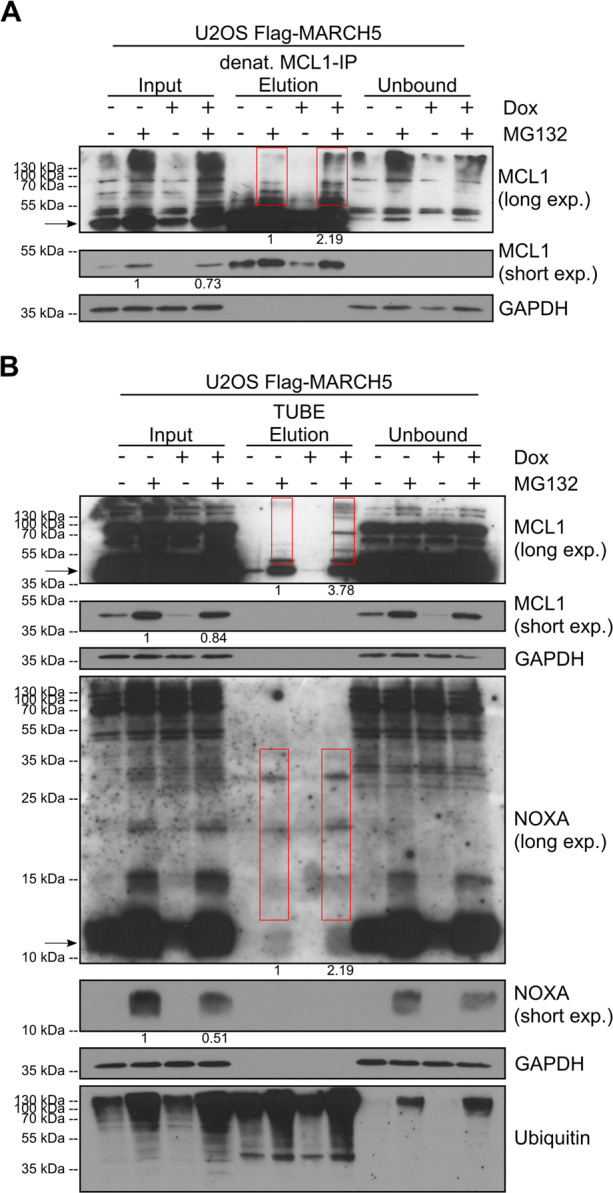
MARCH5 increases the rate of ubiquitination of both MCL1 and NOXA. **a** Input, elution and unbound fraction of a denaturing immunoprecipitation of MCL1 in U2OS Flag-MARCH5 overexpressing cells were analyzed by immunoblot. Doxycycline treatment for 24 h was used to induce overexpression of Flag-MARCH5 and MG132 treatment for 2 h to enrich for ubiquitinated proteins. For MCL1 a short and a long exposure are shown. Numbers below the blots show the quantification of the respective bands. In the long MCL1 exposure the bands with a higher molecular weight (red boxes) than the unmodified MCL1 signal (arrow) were quantified by normalizing the signal in the red boxes to the respective input signal shown in the short exposure of MCL1. The short exposure itself was normalized to GAPDH and the input with MG132 treatment. **b** Input, elution and unbound fraction of a TUBE assay in U2OS Flag-MARCH5 cells were analyzed by immunoblot. Doxycycline treatment for 24 h was used to induce overexpression of Flag-MARCH5 and MG132 treatment for 2 h to enrich for ubiquitinated proteins. For NOXA and MCL1 a short and a long exposure are shown. In the long exposure, the bands with a higher molecular weight (red boxes) than the unmodified MCL1 or NOXA signal (arrow) were quantified by normalizing the signal to the respective input signal shown in the short exposures. The short exposures themselves were normalized to GAPDH and the input with MG132 treatment.

The question remained whether MARCH5 can ubiquitinate both MCL1 and NOXA or only one of them. Hence, we induced MARCH5 overexpression in U2OS cells and used the proteasome inhibitor MG132 to enrich for ubiquitinated proteins. Next, we performed a MCL1 immunoprecipitation under denaturing conditions. Indeed, more and stronger higher molecular weight bands were detected in the MARCH5 overexpressing cells (Fig. 7a, red boxes), indicating a higher extent of MCL1 ubiquitination. We also used the same experimental setup of MARCH5 overexpression and MG132 treatment to use TUBEs (Tandem Ubiquitin Binding Entities) for the pulldown of endogenous ubiquitinated proteins. As before, MCL1 showed a higher number of high molecular weight bands when MARCH5 was overexpressed, (Fig. 7b, red boxes). Critically also NOXA showed a relative increase in higher molecular weight bands, suggesting that the rate of NOXA protein ubiquitination is higher after MARCH5 overexpression. This observation is consistent with the idea that MARCH5 ubiquitinates NOXA/MCL1 complexes.

### NOXA primes cells for apoptosis that undergo slippage

**Fig. 8:**
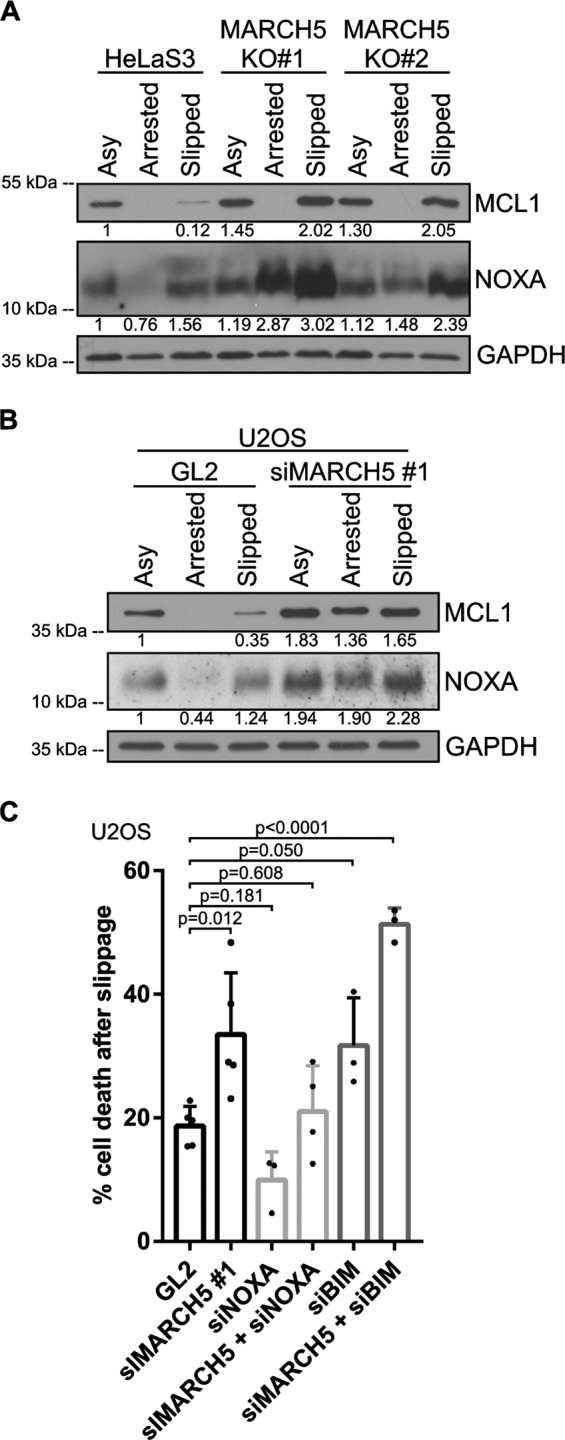
MARCH5 sensitizes cells to apoptosis upon slippage. **a** Parental HeLaS3 cells and two independent MARCH5-KO clones were either left asynchronous (Asy) or synchronized by a double thymidine block and released into paclitaxel. Once the mitotic index reached about 30%, mitotic cells were shaken off and re-plated in the same medium in a fresh dish. 10 h later another shake off was performed and all detached cells were harvested as “Arrested”. Adherent cells were trypsinized and harvested as “Slipped”. Numbers below the blots show the quantification of the respective bands. Quantification was normalized to the GAPDH signal and to the untreated sample (Asy) of the parental HeLa cells. **b** U2OS cells were transfected with a control siRNA (GL2) or a siRNA targeting MARCH5. Cells were otherwise treated like in a). Quantification was normalized to the GAPDH signal and to the untreated sample (Asy) of the U2OS cells transfected with the control siRNA (GL2). **c** Analysis of the percentage of U2OS cells that died in interphase after slipping out of mitotic arrest. U2OS cells were transfected with the indicated siRNAs, synchronized with a single thymidine block, released into paclitaxel and followed by live cell imaging for 72 h. 100 cells entering mitotic arrest were assessed, but only cells that slipped were taken into account for this analysis. All data displayed are mean ± s.d. of five (GL2, siMARCH5), four (siNOXA + siMARCH5), or three (siNOXA, siBIM, siBIM + siMARCH5) independent experiments, indicated as dots. One-way ANOVA, followed by the Holm–Sidak’s multiple comparisons test was performed with *p* < 0.05 considered as statistically significant.

We initially speculated that caspase activation may enhance NOXA-mediated apoptosis in a feed-forward loop by limiting MCL1/NOXA interaction due to caspase-mediated cleavage of MCL1, generating a pool of free NOXA that may neutralize other survival proteins [37, 38]. While we never found any evidence of MCL1 cleavage during mitotic arrest, we noted that the presence of free NOXA no longer sequestered by MCL1 under these conditions (Supplementary Fig. 4a). Disappointingly, but maybe not surprising, we failed to find evidence for NOXA interaction with BCL2 or BCLX to corroborate our initial hypothesis (Supplementary Fig. 4b). We thus considered that accumulation of NOXA after loss of MCL1 might serve to prime cells that may escape mitotic death due to slippage, as MCL1 expression levels are restored in G1 phase [15, 16]. Hence, we investigated how depletion of MARCH5 affects the balance of BCL2-protein family members in cells that undergo slippage. In parental HeLa cells MCL1 levels drastically decreased and slowly recovered in slipped cells, while NOXA levels were high (Fig. 8a). In contrast, MARCH5 knockout cells showed strongly increased levels of both MCL1 and NOXA after slippage. Similar effects were observed in U2OS cells after knockdown of MARCH5 (Fig. 8b). We therefore were wondering if the survival of cells that underwent slippage is also influenced by MARCH5. We analyzed cells that slipped out of mitotic arrest during our live cell experiments and assessed the percentage of cells that died in the following interphase. This short-term analysis of cells that underwent slippage revealed that the rate of cell death in the interphase following mitotic arrest was indeed increased by the lack of MARCH5 (Fig. 8c). Importantly, this increase was dependent on NOXA, since slippage cells depleted of MARCH5 and NOXA, but not BIM, had a cell death rate comparable to control cells (Fig. 8c). Together this suggests that the accumulation of NOXA after MCL1 depletion during mitotic arrest in cells lacking MARCH5 primes cells for apoptosis in the next interphase.

## Discussion

Here, we show for the first time that the mitochondrial E3-ligase MARCH5 plays an important role for cell survival during extended mitotic arrest and after mitotic slippage. Both the mitotic cell death sensitive HeLa cells as well as the more slippage-prone U2OS or A549 cells can be sensitized to mitotic inhibitor treatment when MARCH5 is depleted. Loss of MARCH5 leads to higher basal levels and increased stability of the pro-survival protein MCL1 as well as the proapoptotic protein NOXA, both key regulators of cell fate during mitotic arrest [8, 16, 19].

While the deregulation of MCL1/NOXA turnover is a likely explanation for the increased cell death susceptibility of MARCH5 deficient cells we cannot exclude that disruption of mitochondrial dynamics by deregulation of MID49, another MARCH5 target, decreases cellular fitness. However, double-knockout of MARCH5 and MID49 was shown to restore normal mitochondrial morphology but unable to fully rescue sensitization of HCT116 to various cell death stimuli [23]. Moreover, our siRNA experiments provide evidence that under conditions of MARCH5 depletion only loss of NOXA, but not BIM, decreases cell death rates to levels seen in control cells expressing MARCH5 and exposed to paclitaxel (Fig. 5b). Admittedly, we cannot fully rule out a potential contribution by other BH3-only proteins, but the protein levels of BID and PUMA [23] were also found unaffected by MARCH5-KO. Together, this suggests that the BH3-only proteins BIM, PUMA, and BID are dispensable for the cell death sensitization caused by MARCH5 deficiency.

We also made some unexpected observations during mitotic arrest in HeLa cells lacking MARCH5; MCL1 is efficiently cleared during the later stages of mitotic arrest in those cells even though our CHX experiments showed that MCL1 is more stable during early mitotic arrest. Intriguingly, once cell death is blocked in HeLa cells MCL1 becomes distinctly more stable. In line with this observation is that in U2OS and A549 cells, which are more resistant to mitotic death to begin with, MCL1 levels are clearly more stable in MARCH5-KO cells than in WT with the remaining degradation of MCL1 correlating with the extent of cell death. This argues that the observed MCL1 degradation in HeLa and partially seen in U2OS cells is enhanced by either direct caspase cleavage or the stop in protein translation induced by caspase-mediated cleavage of eukaryotic initiation factors [39]. However, we never observed the accumulation of fragments of caspase-processed MCL1 which carries caspase-sensitive motives at position 124 and 154 [40, 41]. This argues that inhibition of protein translation by caspase-mediated cleavage of specific translation initiation factors like eIF4G [42] and eIF4B [43] contributes to depletion of MCL1 in cells undergoing mitotic cell death.

In contrast to MCL1, NOXA levels remain high throughout mitotic arrest in all MARCH5 deficient cells tested. Remarkably, once cell death is blocked in HeLa cells, NOXA levels drop drastically in early mitotic arrest (Fig. 6a, b). This suggests that MCL1 levels dictate NOXA half-life and that NOXA can accumulate more easily when MCL1 is depleted or neutralized by other means. However, while the levels of NOXA remain relatively high throughout mitotic arrest in MARCH5 deficient cells we also noted that NOXA levels appear to increase in the late stages of mitotic arrest, at least in HeLa, U2OS, and HCT116 cells. This was unexpected, especially in HeLa cells, since near-complete PARP1 cleavage indicated that apoptosis had been initiated in the majority of cells. This raised the possibility that increased NOXA levels may prime cells to apoptosis that potentially escaped mitotic arrest by slippage. Such cells carry the danger of becoming polyploid when failing cytokinesis or becoming genomically instable after chromosome missegregation. Hence it seems to make sense to prime such cells for cell death. Consistently, NOXA depletion reduced the rate of cell death after slippage (Fig. 8c).

We could also establish that MARCH5 dependent degradation of MCL1 requires the presence of and binding to NOXA. In contrast, exogenous NOXA levels seen in HCT116-allBCL2KO cells were barely affected by MARCH5 depletion when MCL1 was present (Fig. 4a). A confounding issue might be that exogenous NOXA levels far exceeded endogenous NOXA levels while for MCL1 both exogenous and endogenous levels were comparable. The high NOXA levels, presumably exceeding the binding capacity of MCL1, could have masked any MARCH5 loss-of-function effects. Consistently, exogenous MCL1 and NOXA levels were reduced in the HCT116-allBCL2KO cells when both proteins were co-expressed and able to bind to each other, in line with co-degradation of both proteins [17].

Even though MARCH5 deficiency stabilized MCL1/NOXA during mitotic arrest neither protein was completely protected from (caspase independent) degradation, arguing that other factors are contributing. At least two other E3-ligases were described to specifically target MCL1 for degradation during mitotic arrest: SCF-FBW7 [14] and APC/C [15]. While it was recently suggested that the former affects MCL1 levels mainly outside of mitosis [8] and MCL1 is degraded by the APC/C in a noncanonical manner [20], our study supports the idea that MARCH5 may contribute to MCL1/NOXA turnover in and outside of mitosis. Thereby, MARCH5 regulates the susceptibility of cells to MTA treatment during mitotic arrest and mitotic slippage. Interfering with MARCH5 activity may hence be a suitable strategy to enhance the efficacy of antimitotic drugs, regardless of their mode of action as mitotic blockers or as mitotic drivers, pushing cells into the next G1 phase.

## Supplementary information

Supplementary Figure Legends

Supplementary Figure 1

Supplementary Figure 2

Supplementary Figure 3

Supplementary Figure 4
