# Supplementary material for: MARCH5-dependent degradation of MCL1/NOXA complexes defines susceptibility to antimitotic drug treatment
Source: Cell Death Differ. 2020 Feb 3;27(8):2297–312. doi: 10.1038/s41418-020-0503-6 (PMC7370223; doi:10.1038/s41418-020-0503-6)
Supplement: Supplementary file 1 — Supplementary Figure Legends [file 41418_2020_503_MOESM1_ESM.docx]

**Supplementary Fig 1: MARCH5-KO sensitizes cells to ABT737 and S63845**

1. Parental HeLaS3 and two independent HeLaS3 MARCH5-KO clones were treated with S63845 to inhibit MCL1 or ABT737 to inhibit BCL2 and BCLX. Live cell imaging was immediately started after treatment. Addition of propidium iodide to the medium allowed the automated assessment of dead cells using the IncuCyte software. A non-linear regression curve of the percentage of dead cells in relation to the total area covered by the cells present at the beginning of the imaging of three independent experiments is shown. The mean and s.d. of exemplary data points is also shown.
2. Same as in a). Three independent experiments with U2OS cells and two U2OS MARCH5-KO bulks genereted with two different guide RNAs.
3. Same as in a). Two independent experiments with A549 cells and two A549 MARCH5-KO bulks genereted with two different guide RNAs.

**Supplementary Fig 2: Knockdown of MCL1 in U2OS and A549 MARCH5-KO bulks does not cause spontaneous cell death**

1. Parental U2SO and two independent U2OS MARCH5-KO bulks were transfected with either control siRNA targeting luciferase (GL2), NOXA and GL2 siRNA (siNOXA), MCL1 and GL2 siRNA (siMCL1) or NOXA and MCL1 siRNA (DKD) for 48 h. Cells were then harvested and prepared for immunoblot analysis. For caspase 3 the full length (fl) and and the position of the cleaved (cl) form is shown.
2. Same as in a) with A549 cells and two A549 MARCH5-KO bulks genereted with two different guide RNAs**.**

**Supplementary Fig 3: Lack of MARCH5 sensitizes HeLa cells to mitotic arrest which is dependent of NOXA but not BIM**

1. HeLaS3 were transfected with either control siRNA targeting luciferase (GL2), GL2 and MARCH5 siRNA (siMARCH5 #1), GL2 and NOXA siRNA (siNOXA) or NOXA and MARCH5 #1 siRNA, left asynchronous (Asy) or synchronized by double thymidine block, released into paclitaxel and harvested in the indicated stages. Numbers below the blots show the quantification of the cleaved PARP signal (arrow). Quantification was normalized to the GAPDH signal and to the M+10 h sample of the HeLa cells transfected with only GL2.
2. Cell fate profiles of HeLaS3 cells arrested in mitosis. Each bar indicates the duration of mitotic arrest of a single cell. Numbers to left of the fate profiles indicated how many cells died or underwent slippage at the end of mitotic arrest. HeLaS3 cells were transfected with the indicated siRNAs, synchronized by a single thymidine block followed by the release into paclitaxel and the start of live cell imaging
3. Box blots with 5-95 % whiskers of the mitotic duration of HeLa cells that died during mitotic arrest (black bars in fate profiles) shown in b). One-way ANOVA was used with Holm-Sidak’s multiple comparison to test the mitotic durations of the siMARCH5 sample against all other knockdowns. The p-values for selected populations are shown with p<0.05 considered statistically signifciant.

**Supplementary Fig 4: MARCH5 deficiency leads to a free pool of NOXA no longer bound by MCL1**

1. Input, elution and unbound fraction of a MCL1 immunoprecipitation in HeLaS3 cells were analysed by immunoblot. HeLaS3 cells were transfected with control siRNA (GL2) or siRNA against MARCH5 (siM5), synchronized with a double thymidine block, released into paclitaxel and mitotic cells were prepared for immunoprecipitation once the mitotic index reached about 30 % (M) or 10 h later. For a NOXA a short and a long exposure are shown.
2. NOXA, MCL1 and MARCH5 immunoprecipitation in HeLaS3 cells were analysed by immunoblot. HeLaS3 cells were synchronized with a double thymidine block, released into paclitaxel and mitotic cells were prepared for immunoprecipitation once the mitotic index reached about 30 %.
