# Supplementary figures and images for: MARCH5-dependent degradation of MCL1/NOXA complexes defines susceptibility to antimitotic drug treatment
Source: Cell Death Differ. 2020 Feb 3;27(8):2297–312. doi: 10.1038/s41418-020-0503-6 (PMC7370223; doi:10.1038/s41418-020-0503-6)

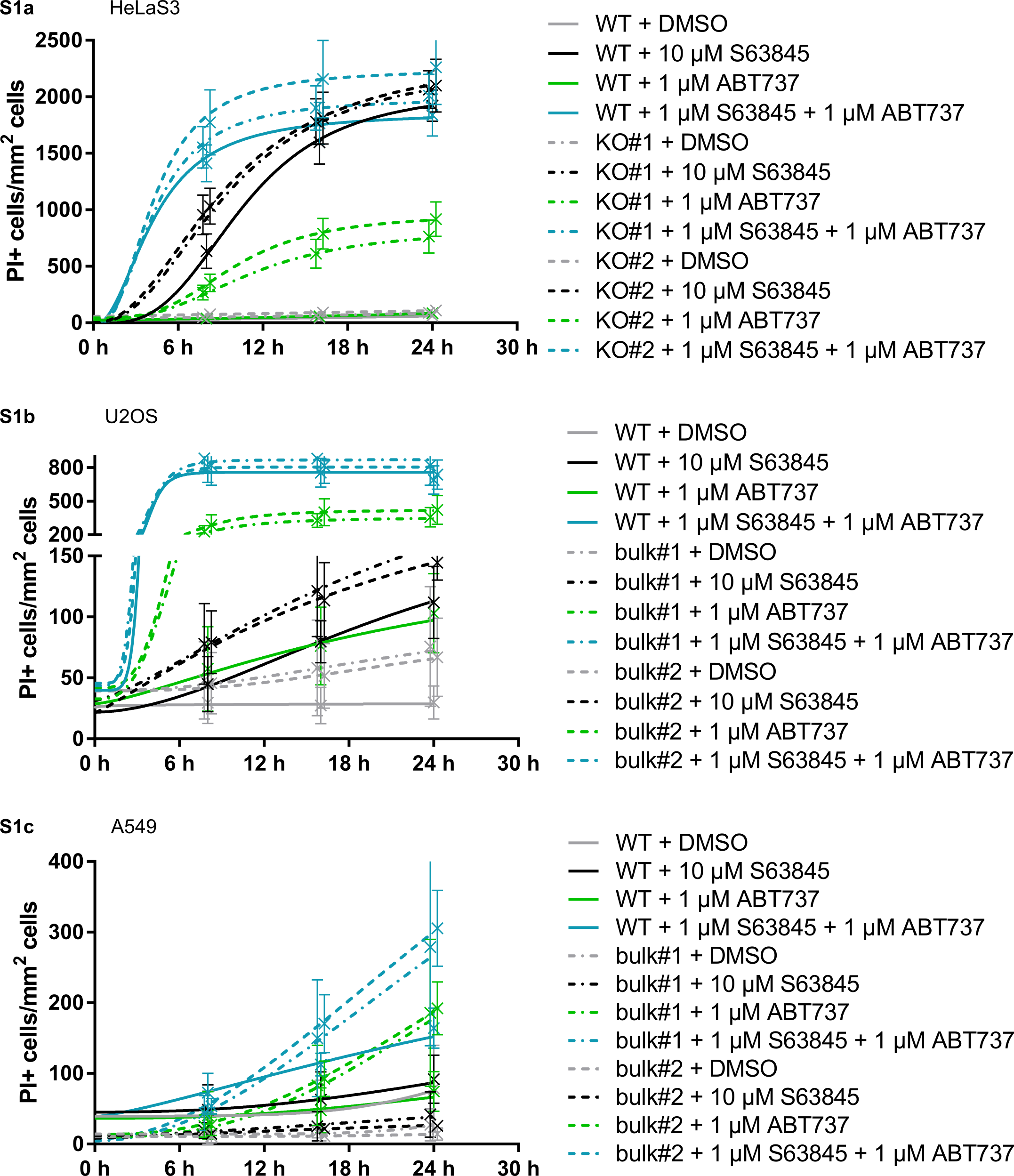

Supplement: Supplementary file 2 — Supplementary Figure 1 [file 41418_2020_503_MOESM2_ESM.png]

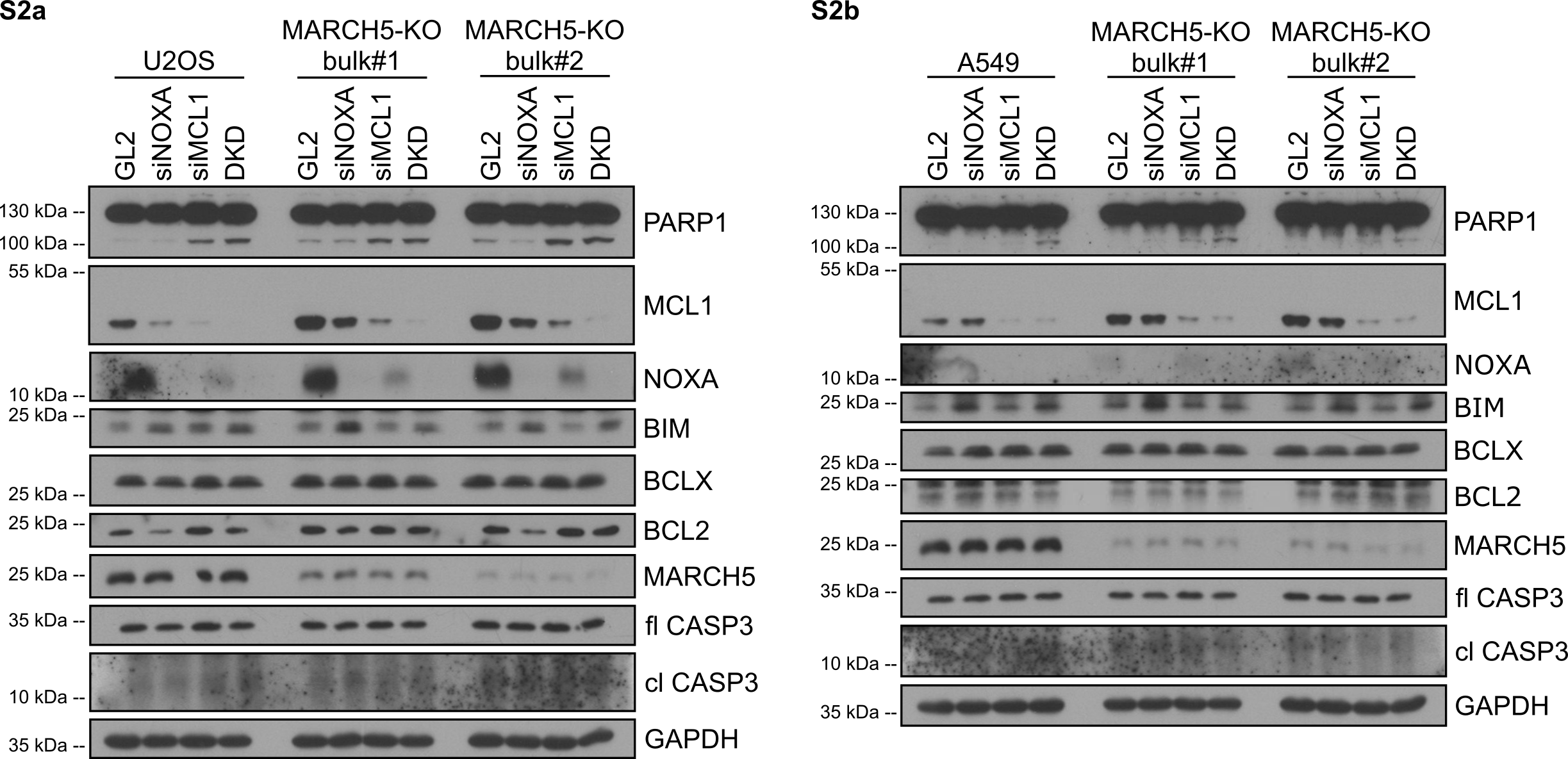

Supplement: Supplementary file 3 — Supplementary Figure 2 [file 41418_2020_503_MOESM3_ESM.png]

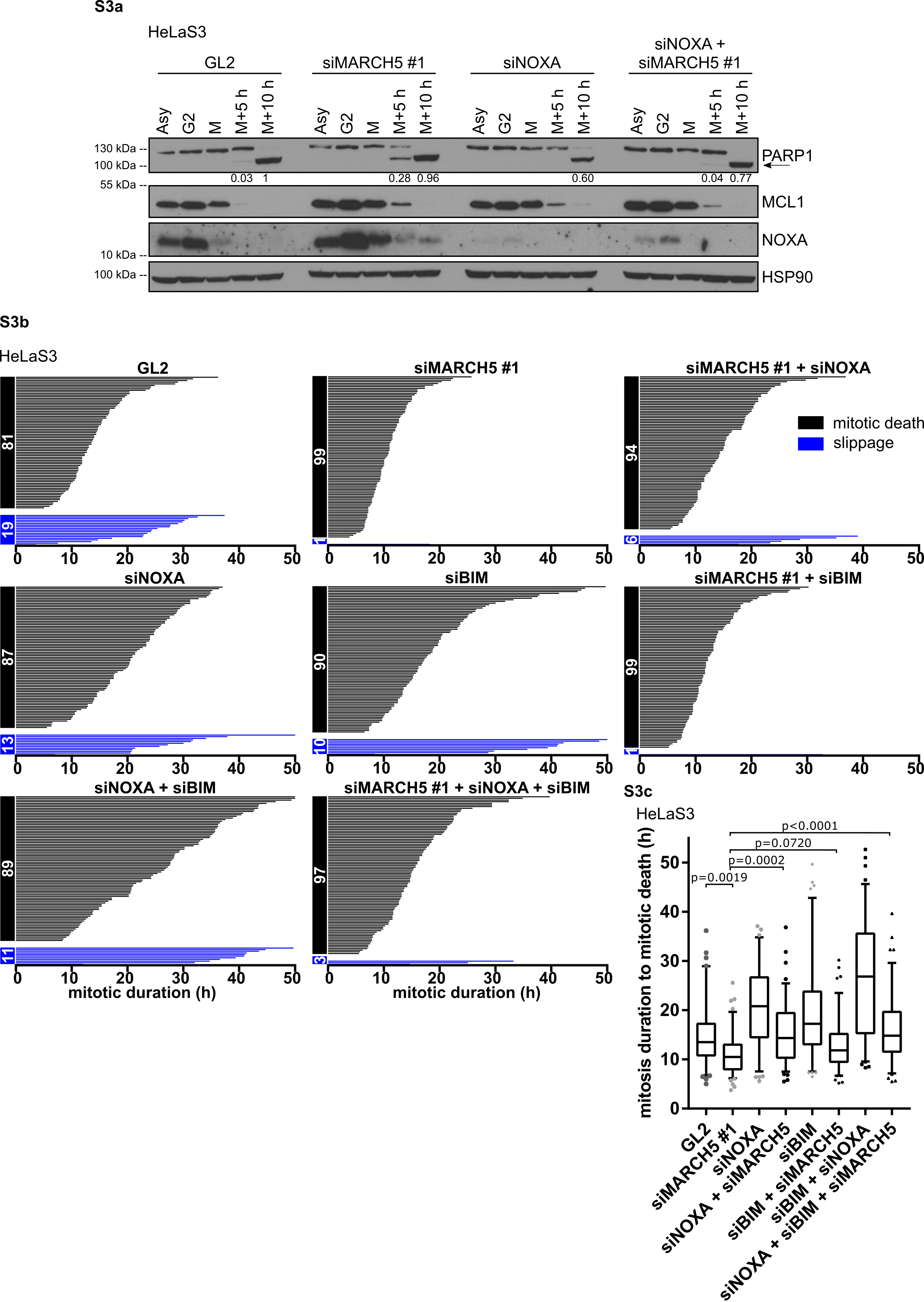

Supplement: Supplementary file 4 — Supplementary Figure 3 [file 41418_2020_503_MOESM4_ESM.png]

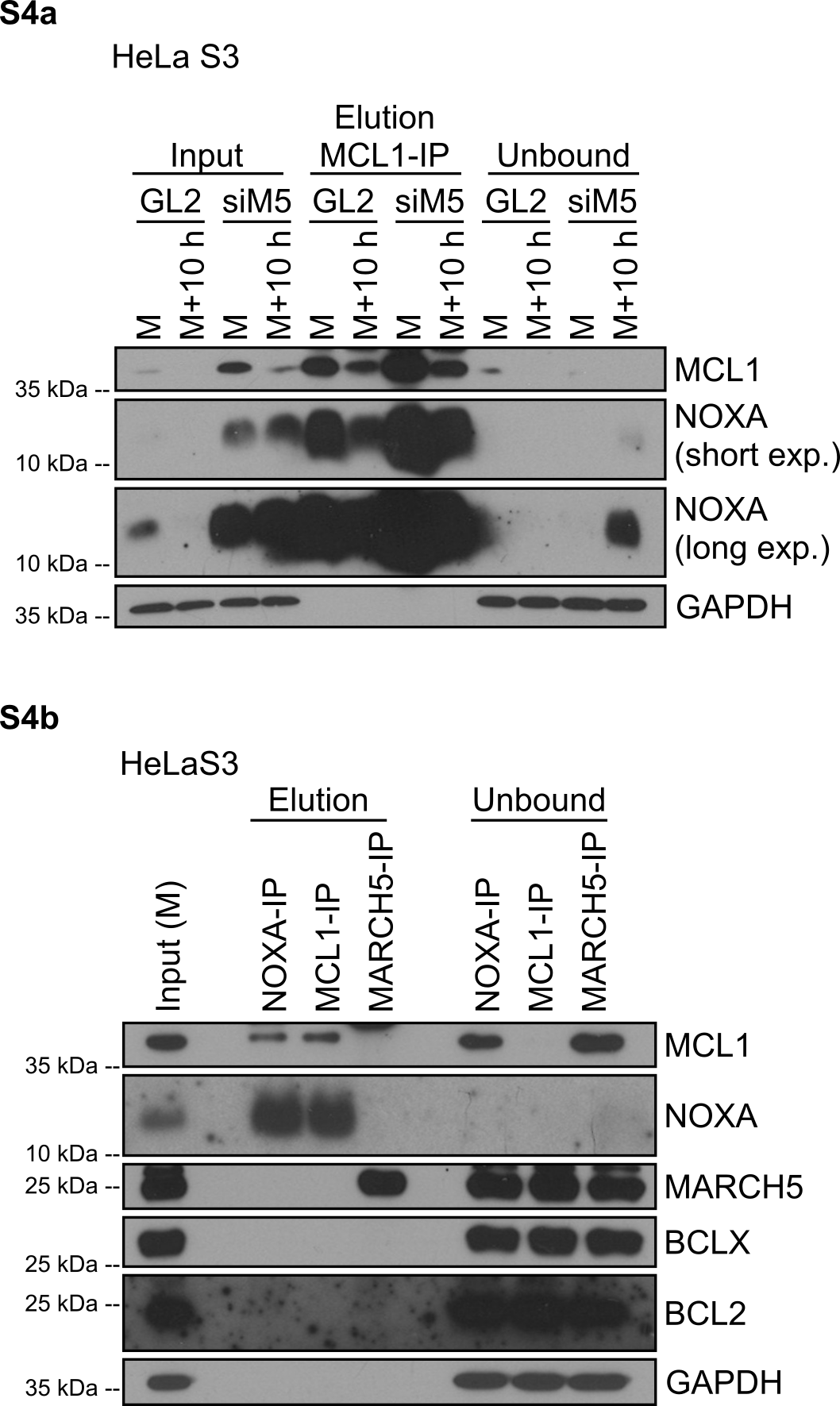

Supplement: Supplementary file 5 — Supplementary Figure 4 [file 41418_2020_503_MOESM5_ESM.png]
